# 中国省区水平肺癌死亡率估计方法研究

**DOI:** 10.3779/j.issn.1009-3419.2011.02.03

**Published:** 2011-02-20

**Authors:** 媛秋 李, 敏 代, 元立 陈, 思维 张, 万青 陈, 珍 代, 小农 邹

**Affiliations:** 1 100021 北京，北京协和医学院 Peking Union Medical College, Beijing 100021, China; 2 100021 北京，中国医学科学院肿瘤医院肿瘤研究所 Cancer Hospital & Institute, Chinese Academy of Medical Sciences, Beijing 100021, China; 3 100021 北京，全国肿瘤防治研究办公室 National Ofce for Cancer Prevention and Control, Beijing 100021, China

**Keywords:** 肺肿瘤, 死亡率, 统计模型, 中国, Lung neoplasms, Mortality, Statistical models, China

## Abstract

**背景与目的:**

目前我国肿瘤登记报告系统不健全，缺乏各省肿瘤流行的基础数据。本文利用我国现有恶性肿瘤死亡资料建立肺癌死亡率统计模型，对2008年我国31个省、自治区、直辖市的肺癌死亡率和死亡人数进行估计。

**方法:**

依据各省市区死亡资料的人口覆盖范围和准确性，建立拟合模型，即直接利用资料、利用历史和现况资料建模推算死亡率变化趋势及利用构成比和恶性肿瘤合计死亡率资料推算部位别死亡率。

**结果:**

2008年我国肺癌死亡病例数为493, 348人，其中男性为338, 346人，女性为155, 002人。按世界人口标化率计，男性和女性肺癌死亡率最高的省份均为吉林（男性52.29/10万，女性24.68/10万），死亡率最低的省份男性为天津（24.12/10万），女性为重庆（8.72/10万）。

**结论:**

研究建立了我国省区水平的肺癌死亡率估计模型，对全国31个省市区肺癌死亡率进行了估计，这些数据将对掌握各地肺癌流行情况提供依据，也为各省区估计其它恶性肿瘤的死亡率提供参考。

## Abstract (en)

**Background and objective:**

At present, population-based cancer registries were not well established and epidemiologic data for cancer were limited at the province level in China. Using the available mortality data for cancer, estimation model of lung cancer was established to provide the mortality profile for the population in 31 provincial regions in China in 2008.

**Methods:**

Based on the population coverage and accuracy of the available mortality for provinces, the methods used to produce mortality estimates fall into 1 of the 3 categories: data were used directly; a model was developed to calculate annual change of mortality rate based on historical and current data; relative frequency data combining with mortality for all sites were used to estimate site-specific mortality.

**Results:**

The total estimated deaths of lung cancer in 2008 were 493, 348 (338, 346 in males and 155, 002 in females) in the whole country. The age-standardized rates were highest in Jilin (52.29 per 100, 000 in males and 24.68 per 100, 000 in females), and the lowest rates were in Tianjin (24.12 per 100, 000 in males) and Chongqing (8.72 per 100, 000 in females).

**Conclusion:**

The models for lung cancer mortality at the province level have been established and the data will provide valuable scientific reference for describing the epidemic of lung cancer as well as estimating other site-specific cancer mortality in China.

全国第三次死因回顾调查报告显示，肺癌是我国居民第1位肿瘤死因，占恶性肿瘤分类构成的22.7%^[1]^。肺癌死亡率在近30年来上升了464.84%^[2]^，成为我国增长幅度最大，危害最为严重的恶性肿瘤。因此，肺癌的防治十分重要^[3]^。但是，由于我国当前肿瘤防控体系很不健全，死因登记报告和肿瘤登记报告系统尚不完善，各地在评估癌症诊治水平、配置卫生资源、制定癌症防治策略时，缺乏详细的基础数据。

本文参照世界卫生组织国际癌症研究中心(World Health Organization, WHO/International Agency for Research on Cancer, IARC)对全球各国癌症流行趋势分析的建模方法，利用我国现有恶性肿瘤死亡数据，结合统计年鉴和人口抽样资料，根据各省资源的充分程度，建立相应统计模型，分析省区水平的恶性肿瘤死亡谱，对2008年我国31个省、自治区、直辖市的肺癌死亡率和死亡人数进行估计，为掌握肺癌死亡的地区与人群分布特征，制定肿瘤预防和控制规划提供科学依据。

## 1 材料与方法

### 1.1 资料来源

① 卫生部公布的全国三次死因回顾调查的恶性肿瘤死亡数据^[1, 4, 5]^(建立模型)；②上海市肿瘤研究所和上海市疾病预防控制中心出版物《上海市区恶性肿瘤发病率、死亡率和生存率(1973-2000)》^[6]^(模型验证)；③国务院人口普查办公室和国家统计局编撰的中国2005年1%人口抽样资料和中国统计年鉴(2009)31个省、市、自治区2008年人口数据^[7, 8]^(建立模型)；④中国卫生统计年鉴(2009)中海南和重庆2008年全死因死亡率和有关恶性肿瘤构成比文献资料^[9-12]^(建立模型)。

### 1.2 统计方法

#### 1.2.1 建立模型

按照IARC全球癌症报告(GLOBOCAN 2008)^[13]^对全球182个国家和30个地区27种肿瘤的死亡、发病和患病估计建模原则，根据各省(市区)死亡资料的质量和获得程度，建立3类估计模型，即直接利用资料(模型A)、利用历史和现况资料建模推算死亡率变化趋势(模型B)、利用构成比和恶性肿瘤合计死亡率资料推算部位别死亡率(模型C)(表 1)。

**1:** 各省市区肺癌死亡率估计模型 Estimates of mortality of lung cancer for provincial regions in China

Model	Provincial region	Condition	Notes
A	Beijing, Shanghai, Tianjin	When mortality rate for the entire population deriving from good quality cancer registry were available	Used directly
B	East: Hebei, Liaoning, Jiangsu, Zhejiang, Fujian, Shandong, Guangdong; Middle: Heilongjiang, Jilin, Shanxi, Anhui, Jiangxi, Henan, Hubei, Hunan; West: Inner Mongolia, Guangxi, Sichuan, Guizhou, Yunnan, Tibet, Shaanxi, Gansu, Qinghai, Ningxia, Xinjiang	When current data were for only a very small component of the population or less representative	The first national mortality survey→Mortality for 2008 using Arithmetic Progression or Geometric Progression method.
C	Hainan, Chongqing	No data	Relative frequency

##### 1.2.1.1 直接利用资料(模型A)

具有全人群肿瘤登记数据的直接利用该登记数据。

##### 1.2.1.2 利用历史和现况资料建模推算死亡率变化趋势(模型B)

对于登记数据仅覆盖部分人口而不具代表性的建模推算。根据1973年-1975年到2004年-2005年期间各地区(东、中、西)分年龄及性别的肺癌死亡率年均变化值及第一次死因调查肺癌死亡数据进行估计。

推算方法：①假定分地区、年龄和性别的肺癌死亡率遵循算术级数变化(Arithmetic Progression method)，估计该死亡率年均增长值和期望死亡率，即

1^[14]^ $
{\rm{r}} = \left( {{P_n} - {P_0}} \right)/n
$

2^[15]^ $
{\rm{E}}\left[{{{\rm{M}}_{{\rm{it}}}}} \right] = {{\rm{ \mathsf{ α} }}_{\rm{i}}} + {{\rm{ \mathsf{ β} }}_{\rm{i}}}{\rm{t}}
$

其中*P_n_*是第三次死因调查分地区、年龄和性别的死亡率，*P*_0_是第一次死因调查相应死亡率，*r*是年均增长值，E[M_it_]指第i个年龄组在第t年的期望死亡率，α_i_是该年龄别基线死亡率，β_i_即公式(1)计算的年龄别年均增长值。

② 假定分地区、年龄和性别的肺癌死亡率变化遵循几何级数增长(Geometric Progression method)，按照下列公式估计死亡率年均增长率和期望死亡率：

3^[14]^ $
r = \sqrt[n]{{{P_n}/{P_0}}} - 1
$

4 $
{P_t} = {P_0}{\left( {1 + r} \right)^t}
$

其中*P_t_*是期望年份的死亡率，*r*是年均增长率。

##### 1.2.1.3 利用构成比和恶性肿瘤合计死亡率资料推算部位别死亡率(模型C)

对于第一次死因调查时尚未设立省级行政区的采用"构成比"方法，用恶性肿瘤合计死亡率推算部位别死亡率^[13]^，包括海南和重庆。

#### 1.2.2 模型验证

① 选择个别省(市区)死因监测或肿瘤登记肺癌死亡率与模型B结果比较，以差值百分比最小为原则进行模型筛选^[16]^；②用上述筛选模型，拟合上海1973年-2000年肺癌死亡率变化趋势，估计2006年上海肺癌死亡率；另根据上海1973年-2000年5个时段肺癌死亡率的线性趋势外推得到上海2006年肺癌死亡率^[17]^；两者与2006年肿瘤登记数据比较进行模型验证。

#### 1.2.3 统计指标

采用1982年中国标准人口和Segi's世界标准人口^[18]^构成分别计算中国人口标化率和世界人口标化率。分性别和年龄别(0岁-14岁，15岁-34岁，35岁-54岁，55岁-74岁，75岁以上)对各省肺癌的死亡率和死亡人数进行估计。东中西部地区的划分根据第三次死因回顾调查采纳的国家统计局标准^[1]^。

## 2 结果

### 2.1 模型验证

按照模型B估算甘肃省2003年和河南省2001年肺癌死亡率，与文献报告甘肃省20个疾病监测点^[19]^及河南14个肿瘤登记点^[20]^同期肺癌死亡率比较，用算术级数法拟合获得的肺癌死亡率差值百分比在3.1%和53.6%之间，均高于文献报告值；用几何级数法拟合结果差值百分比在-36.2%和-65.2%之间，均小于文献报告值(表 2)。用算术级数法拟合模型结果，除河南省女性标化死亡率以外(差值百分比分别为43.9%和-36.2%)，均优于几何级数法。

**2:** 模型拟合与文献报告的肺癌死亡率比较 Comparison of mortality rates between literature and estimated data produced by two models for lung cancer

Province	Gender	Item	Mortality rate (1/100, 000)				Difference (%)	
			Literature report	Model 1	Model 2		Model 1-Literature	Model 2-Literature
Gansu (2003)	Both sexes	Crude	12.00	16.25	4.76		35.4	-60.4
		ASR	12.02	14.09	4.19		17.2	-65.2
	Male	Crude	14.92	22.91	5.66		53.6	-62.1
		ASR	N	20.29	5.01		N	N
	Female	Crude	8.96	9.24	3.66		3.1	-59.2
		ASR	N	7.85	3.20		N	N
Henan (2001)	Both sexes	Crude	21.03	26.87	8.65		27.8	-58.9
		ASR	13.77	20.17	7.29		46.5	-47.1
	Male	Crude	27.48	35.01	10.68		27.4	-61.2
		ASR	19.44	28.73	9.14		47.8	-53.0
	Female	Crude	14.10	17.73	6.43		25.8	-54.4
		ASR	8.38	12.06	5.35		43.9	-36.2
Crude: the crude mortality rate; ASR: the age-standardized mortality rate of Chinese population in 1982; Model 1: Arithmetic Progression method; Model2 : Geometric Progression method; Difference (%): (Estimated value-Reported value)/Reported value× 100; N: No report.								

用算术级数法计算的分地区、年龄和性别肺癌死亡率年均变化值略高于用上海1973年-2000年5个时段肺癌死亡率估算的结果(表 3)，提示上海市肺癌年龄别死亡率变化速度小于全国特别是东部地区。按模型B估计的2006年上海市男女合计、男性和女性的肺癌死亡率分别为20.8/10万、34.6/10万和10.6/10万，介于线性趋势外推值(23.4/10万、36.1/10万和12.6/10万)和肿瘤登记报告值(17.9/10万、26.7/10万和10.2/10万)之间，验证了模型B的可行性。

**3:** 肺癌死亡率年均变化值及模型验证结果 The average changes by year and model validation of estimated age-standardized mortality for lung cancer

Item	Period	Indicator	Both sexes						Male						Female				
			0-14	15-34	35-54	55-74	75^+^		0-14	15-34	35-54	55-74	75^+^		0-14	15-34	35-54	55-74	75^+^
Change per year (1/100, 000)	1973-2005	East	0	0	0.3	3.0	9.9		0	0	0.4	4.1	14.4		0	0	0.2	1.8	6.6
		Middle	0	0	0.4	3.6	10.8		0	0	0.5	5.0	16.0		0	0	0.2	2.1	6.9
		West	0	0	0.3	2.6	7.0		0	0	0.4	3.8	10.5		0	0	0.2	1.4	4.1
	1973-2000	Shanghai	0	0	-0.6	0.2	9.0		0	0	-1.1	0.5	16.3		0	0	-0.2	0	2.9
ASR of baseline (1/100, 000)	1973^*^	Shanghai (R)	0	0.5	19.0	161.6	227.9		0	0.4	25.4	240.0	402.2		0	0.5	12.5	88.5	137.7
	1973^**^	Shanghai (N)	0	0.7	15.8	140.8	188.0		0	0.7	21.9	219.0	349.3		0	0.6	9.9	72.5	112.8
Estimated/observed ASR(1/100, 000)	2006	Shanghai (Extrapolation)	23.4						36.1						12.6				
	2006	Shanghai (Model)	20.8						34.6						10.6				
	2006	Shanghai (Observed)	17.9						26.7						10.2				
ASR: the age-standardized mortality rate of Chinese population in 1982; Shanghai (R): data from Shanghai registry; Shanghai (N): data from the first national mortality survey; Shanghai (Extrapolation): the age standardized mortality rate (ASR) estimated by extrapolation of Shanghai registry data; Shanghai (Model): the age standardized mortality rate (ASR) estimated by model B; ^*^: the average rate in 1973-1977 from Shanghai registry; ^**^: the average rate in 1973-1975 for Shanghai in the first national mortality survey.																			

### 2.2 肺癌死亡率及死亡人数估计

按粗死亡率计，2008年我国31个省、市、自治区肺癌死亡率最高的为上海(男性76.49/10万，女性35.82/10万)，最低的为西藏和宁夏(西藏男性最低：25.14/10万，宁夏女性最低：12.09/10万)(表 4，图 1)。按中国人口标化率计，死亡率处于高水平的有海南(男性43.71/10万，女性25.52/10万)、吉林(男性39.05/10万，女性18.50/10万)和黑龙江(男性36.75/10万，女性16.78/10万)，较低水平的是甘肃(男性22.72/10万，女性8.77/10万)和广西(男性22.87/10万，女性8.78/10万)。按世界人口标化率计，男性和女性肺癌死亡率最高的均为吉林(男性52.29/10万，女性24.68/10万)，最低的男性为天津(24.12/10万)，女性为重庆(8.72/10万)。各省肺癌死亡人数差别很大，男性最低的为西藏(355人)，超过2万人的分别有山东(26, 949人)、河南(24, 874人)、江苏(23, 294人)和四川(20, 905人)；女性低于1, 000人的包括西藏(181人)、青海(341人)和宁夏(370人)，高于1万人的包括山东(14, 239人)、江苏(11, 968人)和河南(11, 786人)。31个省、市、自治区合计肺癌死亡病例数为493, 348人，其中男性为338, 346人，女性为155, 002人。

**4:** 2008年我国各省市区估计肺癌死亡率(1/10万) Estimated mortality rate of lung cancer in China during the year 2008(per 100, 000)

Provincial region	Both sexes				Male				Female		
	Crude	ASR^1^	ASR^2^		Crude	ASR^1^	ASR^2^		Crude	ASR^1^	ASR^2^
Beijing	45.04	16.90	23.47		55.97	22.17	30.90		33.83	12.01	16.67
Tianjin	33.64	14.48	20.00		38.55	17.45	24.12		28.72	11.50	15.88
Hebei	39.69	24.00	32.29		52.49	32.65	44.02		25.59	15.08	20.22
Shanxi	38.09	25.30	34.00		52.77	35.76	48.12		23.05	14.88	20.02
Inner Mongolia	30.17	19.70	26.25		42.83	27.92	37.30		16.62	10.86	14.42
Liaoning	50.65	26.71	35.89		66.09	35.44	47.74		34.71	17.91	24.01
Jilin	47.03	28.84	38.53		62.60	39.05	52.29		30.81	18.50	24.68
Heilongjiang	42.81	26.79	35.92		57.69	36.75	49.37		27.29	16.78	22.47
Shanghai	56.28	17.89	24.92		76.49	26.68	37.27		35.82	10.21	14.40
Jiangsu	45.76	22.24	30.01		61.29	31.28	42.28		29.94	13.71	18.50
Zhejiang	44.46	23.04	31.01		61.68	32.55	43.91		27.53	13.71	18.44
Anhui	43.43	23.06	31.04		60.07	33.05	44.53		26.94	13.58	18.34
Fujian	34.36	20.91	28.15		46.30	29.33	39.56		22.35	12.84	17.30
Jiangxi	35.69	21.88	29.46		48.51	31.00	41.80		22.49	13.08	17.65
Shandong	42.25	22.12	29.79		56.23	31.19	42.10		29.75	14.68	19.74
Henan	38.62	23.61	31.80		51.39	33.62	45.34		24.90	14.10	19.02
Hubei	40.29	21.92	29.54		54.69	31.13	42.00		25.38	13.09	17.69
Hunan	41.55	22.08	29.73		56.93	31.17	42.06		26.12	13.33	17.96
Guangdong	29.00	20.67	27.90		39.11	29.72	40.18		19.16	12.58	17.00
Guangxi	27.14	15.79	21.07		36.82	22.87	30.62		16.23	8.78	11.66
Hainan	43.53	34.81	25.44		51.24	43.71	31.86		34.37	25.52	18.57
Chongqing	23.91	23.17	17.13		35.24	35.51	26.21		12.79	11.83	8.72
Sichuan	34.95	17.10	22.84		49.66	24.48	32.78		20.18	9.75	12.97
Guizhou	25.11	16.29	21.77		34.39	23.29	31.21		15.02	9.32	12.42
Yunnan	24.01	16.90	22.54		33.18	24.72	33.06		13.99	9.24	12.29
Tibet	19.30	15.84	21.20		25.14	22.72	30.56		12.15	9.07	12.05
Shaanxi	30.27	18.06	24.09		41.56	25.42	34.01		18.12	10.54	14.00
Gansu	23.00	15.76	21.05		32.65	22.72	30.44		12.96	8.77	11.67
Qinghai	20.56	16.63	22.16		28.39	23.50	31.40		12.17	9.65	12.84
Ningxia	21.14	17.13	22.90		30.12	24.24	32.46		12.09	9.87	13.18
Xinjiang	22.53	17.44	23.28		32.65	24.87	33.30		12.43	9.78	13.00
Crude: the crude mortality rate; ASR^1^: the age-standardized mortality rate of Chinese population in 1982; ASR^2^: the age-standardized mortality rate of the world standard population.											

**1:**
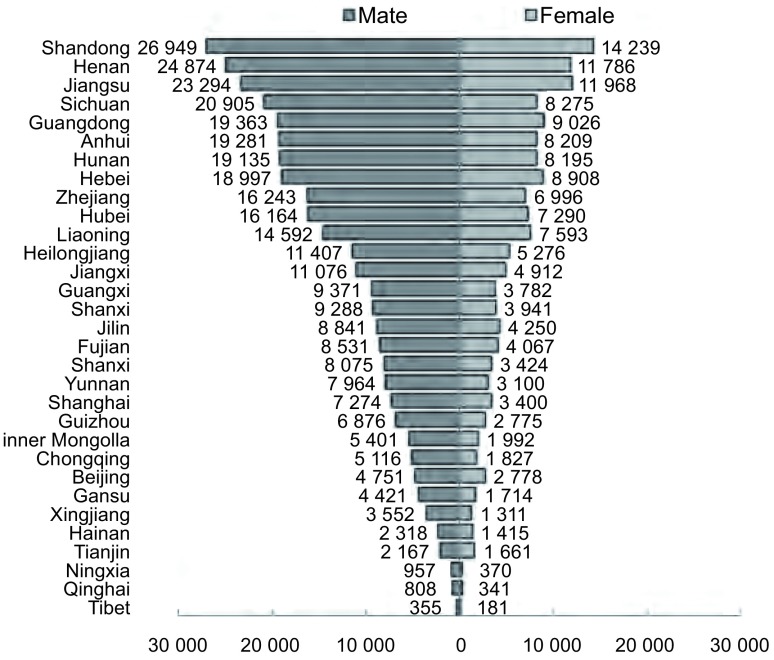
2008年我国各省市肺癌估计死亡数 Estimated numbers of deaths of lung cancer in 2008, by sex and provincial region

## 3 讨论

本研究借鉴WHO/IARC对世界各国癌症负担的估计方法构建模型，利用目前我国最大样本的数据资源，包括全国三次死因调查及人口统计年鉴资料，首次对全国31个省、市、自治区肺癌的死亡率水平进行分析和估计，这些数据将对各地掌握肺癌流行趋势，制定地区性防治策略提供参考。

从估计的肺癌死亡人数来看，2008年我国31个省(市区)合计为49万，略高于Ferlay J^[13]^估计的45万人和陈万青^[21]^2005年估计的47万人。后两者仅采用全国第三次死因抽样调查样本数据进行估计，未考虑各省基础数据的差异。本文利用全国各省肺癌的基础信息，对30年来全国东、中、西部地区肺癌死亡率变化规律进行分析，因此，构建的模型更符合实际情况和反映各地肺癌的死亡水平。用算术级数法对甘肃省和河南省肺癌死亡率的估计结果高于文献报告值，几何级数法则低于文献报告值，但由于常规死因资料的错报和漏报^[22]^，以及肿瘤登记资料人群覆盖率(河南为10.52%)较低，文献报告值并不能作为金标准判定模型拟合的准确性。因此，在现有资料情况下运用算术级数法比几何级数法更为合理。上海1973年-2000年肺癌死亡率拟合模型显示(表 3)，用算术级数法较线性外推结果更接近实际情况，该法对肺癌死亡率可进行较为可靠的估计。

我国大部分省(市区)尚无肺癌统计资料，特别是由于生命统计制度不完善、肿瘤诊疗水平较低、肿瘤死亡率漏报严重等导致人群肿瘤数据缺乏和质量不可靠，使得肿瘤预防和控制的策略制定无本可依。对于仅有70年代死因回顾调查数据的地区，其年代、队列效应，甚至是时间趋势均无法得到准确反映。目前国际上多采纳时间趋势线性模型、年龄-时期-出生队列(Age-Period-Cohort)模型、二元时间序列自回归模型、状态空间模型等进行人群肿瘤发病、死亡负担的估计和预测，但上述方法均需长期连续的数据^[23]^，无法引入本文的估计模型。因此，本研究虽考虑到地区、性别和年龄等影响死亡率的主要因素，充分利用覆盖两次死因调查资料拟合模型，但其估计结果仍会存在一定偏差。

过去30余年除人口年龄结构外，我国的环境、经济状况及医疗卫生服务水平等发生了很大变化，这些改变也会不同程度地影响各地癌症死亡率水平。按中国人口标化率比较，70年代我国肺癌死亡率较高的省份有上海、天津、北京，最低的省份有甘肃、广西、西藏。据本文对2008年死亡率水平的估计，海南、吉林和黑龙江是肺癌死亡率最高的地区，广西、天津和甘肃最低。结合1990年-1992年全国第二次死因调查结果，27个省(市区)中黑龙江、吉林和辽宁死亡率最高，甘肃、湖南和江西最低。各地的肺癌死亡率变化呈现了以下特点：各省肺癌的死亡率变化幅度差异很大，70年代水平较高的地区，其增长幅度较小，导致了死亡率水平排序的后移；而水平较低的地区，排位则可能前移。但从整体来看，仍然是东部较发达地区的肺癌死亡率高，西部边远地区的死亡率较低。由于重庆和海南在第一次死因调查时尚未设立省级行政区，无法在第一次死因调查资料基础之上进行死亡率变化的推算，故采用了准确性较低的"构成比"法。该法依赖于现有文献报告肿瘤全死因构成比和卫生统计年鉴提供的全死因死亡率资料。其中海南省肿瘤全死因构成比(28.64%)高于死因研究结果^[22]^，有可能引起海南省肺癌死亡率过高的估计。

本研究利用全国三次死因调查及人口统计年鉴资料构建统计模型，对我国省(市区)的肺癌死亡率水平进行估计。通过数据回代及与现有文献资料比较，证实能较真实地反映各地肺癌死亡情况。本研究对各地估计其它恶性肿瘤的死亡率提供了一个新思路，尽管研究仍有不足，但在尚未建立较为完善的死因登记报告和肿瘤登记报告系统的地区，利用死因调查资料来估计人群恶性肿瘤死亡水平具有重要的意义。

## References

[1] The Ministry of Health of the People's Republic of China (2008). Third national retrospect pot-check of death-causation.

[2] National Office for Cancer Prevention and Control, National Center for Cancer Registry, Disease Prevention and Control Bureau, MOH (2010). Chinese Cancer Mortality Report-Third national retrospect spot-check of deathcausation.

[3] Dong ZW, Qiao YL, Li LD (2002). Report of Chinese cancer control strategy. Bullet Chin Cancer.

[4] National Office for Cancer Prevention and Control (1980). Investigation of China Cancer Mortality (1973-1975).

[5] National Office for Cancer Prevention and Control (2008). Investigation of China Cancer Mortality (1990-1992).

[6] Gao YT, Lu W (2007). Cancer incidence, mortality and survival rates in urban Shanghai (1973-2000).

[b7] 7Office of 1% Population Sampling Survey and Population and Employment Division of SSB. Data of 1% Population sampling survey in China. http://www.stats.gov.cn/tjsj/ndsj/renkou/2005/renkou.htm.国务院全国1%人口抽样调查领导小组办公室, 国家统计局人口和就业统计司. 2005年全国1%人口抽样调查资料. http://www.stats.gov.cn/tjsj/ndsj/renkou/2005/renkou.htm.

[8] National Bureau of Statistics (2010). China Statistical Yearbook 2009.

[9] The Ministry of Health of the People's Republic of China (2009). Chinese Health Statistics 2009.

[10] Luo JH, Chen Y, Wang HM (2005). Analysis of death cause of residents in monitoring spots in Hainan Province during 2000-2004. China Tropical Medicine.

[11] Zhang YQ, Yi D, Tang GL (2009). Dynamic analysis on major death causes for residents in Chongqing. Chongqing Medicine.

[12] Mao DQ, Feng LG, Pan CB (2009). Epidemiology and variation trend of the malignant tumor mortality among residents of urban in Chongqing municipal. Chongqing Medicine.

[13] Ferlay J, Shin HR, Bray F (2010). Estimates of worldwide burden of cancer in 2008: GLOBOCAN 2008. Int J Cancer.

[14] Yang SQ (1986). Health Statistics.

[15] Dyba T, Hakulinen T (2000). Comparison of different approaches to incidence prediction based on simple interpolation techniques. Stat Med.

[16] Yang L, Parkin DM, Li L (2004). A comparison of the sources of cancer mortality in China. Cancer causes and control.

[17] Ziegler RG, Anderson WF, Gail MH (2008). Increasing breast cancer incidence in China: the numbers add up. J Natl Cancer Inst.

[18] Segi M (1960). Cancer mortality for selected sites in 24 countries (1950-1957).

[19] Ren XL, Ge PF, Wei ZZ (2005). Analysis of deaths from tumors reported from disease surveillance system of gansu province. Disease Surveillance.

[20] Liu SZ, Dai DX, Li G (2002). An analysis of malignant mortality distribution in henan province in 2001. Bullet Chin Cancer.

[21] Chen WQ, Zhang SW, Zou XN (2010). Estimation and projection of lung cancer incidence and mortality in China. Chin J Lung Cancer.

[22] Yang GH (2005). Deaths and their risk factors among Chinese population.

[23] Li YQ, Zou XN (2010). Estimates of the cancer incidence, mortality and prevalence: a systematic review. J Cancer Control and Treatment.

